# Genetic polymorphism of heme oxygenase 1 promoter in the occurrence and severity of chronic obstructive pulmonary disease: a meta‐analysis

**DOI:** 10.1111/jcmm.13028

**Published:** 2016-12-20

**Authors:** Hongbin Zhou, Xiwang Ying, Yuanshun Liu, Sa Ye, Jianping Yan, Yaqing Li

**Affiliations:** ^1^ Department of Respiratory MedicineZhejiang Provincial People's HospitalHangzhouZhejiangChina

**Keywords:** HMOX1, COPD, length polymorphism, susceptibility, severity

## Abstract

Heme oxygenase 1 (HMOX1) plays an important role in the development of chronic obstructive pulmonary disease (COPD). However, the association of *HMOX1* length polymorphism in promoter region to the risk and severity of COPD has not been well studied. In this study, we searched the databases including PubMed, EMBASE, Cochrane Library and China National Knowledge Infrastructure (CNKI) and extracted the information from related articles. Pooled odds ratios (ORs) and 95% confidence intervals (CIs) were calculated to study the effect of *HMOX1* polymorphism on the risk and severity of COPD. As a result, nine studies were included for this meta‐analysis. Higher frequencies of L allele and type I genotype (containing at least one L allele) were found in patients with COPD (for L allele, OR 2.02, 95% CI: 1.32–3.11, *P* = 0.001; for type I genotype, OR 1.82, 95% CI: 1.28–2.61, *P* = 0.001), especially in Asian population (for L allele, OR 2.23, 95% CI: 1.68–2.95, *P* < 0.001; for type I genotype, OR 2.02, 95% CI: 1.51–2.70, *P* < 0.001). Genotyping method, source of control subjects, literature quality and language also affected the results to some extent. However, there was little difference in *HMOX1* genotypes distribution in patients with COPD with different severity. Our study indicated L allele and type I genotype were related to the susceptibility but not the severity of COPD.

## Introduction

COPD is a common airway disease, leading to an increasing mortality and morbidity in the world 1, 2. Despite its high incidence in recent years, the detail mechanism of this disease has not been fully elucidated so far 3. It is widely accepted that the imbalance of oxidation and reduction reaction plays an important role in the development of COPD 4, 5. The overproduction of oxidative substance is attributed to the pathogenesis of COPD.

Heme oxygenase has the ability to resist damage caused by oxidative stress. There are three types of heme oxygenase isozymes 6, 7. Among these, HMOX1 or HO‐1, the inducible isoform responding to various stimuli in the environment, was confirmed to play a pivotal role in protecting against mucus hypersecretion 8, emphysema 9, 10, 11, airway inflammation 12, which were the main characteristics of COPD, in a series of studies. The activity of this gene is dependent on its promoter, and length polymorphism caused by various kinds of GT repeat numbers ((GT)_n_) exists in promoter sequence. In view of the great variety of GT repeat numbers in different populations, these polymorphisms are generally divided into three types of alleles, named S, M and L alleles, representing short, medium and long GT repeat sequence, respectively 13. Different genotypes lead to different activity levels of HMOX1, affect the degree of oxidative stress in the organism and finally influence the susceptibility to COPD. Thus, many researchers focused their attention on the relationship between (GT)_n_ polymorphism and COPD in the past decades. However, they obtained inconsistent results, which might be due to ethnicity, sample size and selection bias 14, 15, 16, 17, 18, 19, 20, 21, 22. Based on these, we conducted this meta‐analysis to determine the association between genetic polymorphism of heme oxygenase 1 promoter and COPD occurrence and severity.

## Materials and methods

### Search strategy

We performed a comprehensive search strategy in several databases including PubMed, EMBASE, Cochrane Library and CNKI to find out the articles about the association between *HMOX1* polymorphisms and COPD. The terms we used as follows: ‘chronic obstructive pulmonary disease’, ‘COPD’, ‘emphysema’, ‘chronic bronchitis’; ‘heme oxygenase1’, ‘hmox1’, ‘ho1’; and ‘genetic polymorphism’, ‘variant’, ‘variation’, ‘association’. Additional studies were identified by a manual search from references of original studies or review articles on this topic. Only studies with full‐text articles published until October 2015 were included.

### Study selection

The criteria for the papers selection were as follows: (*i*) studies with case–control or prospective longitudinal cohort design; (*ii*) studies with at least two comparison groups (COPD *versus* control or less severe COPD patients *versus* more severe COPD patients (measured by lung function)); (*iii*) the study including HMOX1 length polymorphisms (GT repeat number) in COPD cases and controls; and (*iv*) provide the available allele and/or genotype frequency in each group.

### Quality assessment

All the included studies were assessed in three aspects consisting of selection, comparability and exposure, with the use of Newcastle–Ottawa Scale (NOS) 23, which has been widely applied in observational studies. Each study was assigned a score from 0 to 9 points, and higher points meant higher quality.

### Data extraction

The data were carefully extracted from all eligible publications independently by two authors according to the inclusion criteria listed above. Once encountering disagreements, we resolved them by discussions with the third person. The information we extracted from papers contains basic information of study (author, publication year), population (sample size, ethnicity, age, source of control subjects, lung function and smoking status), COPD definition, genotype distribution in cases and controls, genotype identification method, etc.

### Data synthesis

OR and 95% CIs were used to assess the strength of association between *HMOX1* polymorphisms and COPD risk and severity.

Heterogeneity assumption was checked by the Cochrane *Q* test. If *P* value for the *Q* test is over 0.10, we consider that there is lack of heterogeneity. We also used the statistic of *I* ^2^ to detect the degree of heterogeneity, with *I* ^2^ <25%, 25–75% and >75% to represent low, moderate and high degree of inconsistency, respectively 24, 25. In the analysis of pooled data, we used two different models according to the trait of the included studies: If no heterogeneity was found, a fixed effect model was adopted to determine the gene effect or the random effect model was used. Moreover, if heterogeneity across studies existed, subgroup analysis was performed to seek out the source of heterogeneity. Studies were subdivided by ethnicity (Asian *versus* Caucasian), genotyping methods (automated sequencing *versus* PCR‐PAGE), source of control subjects (general population‐based *versus* hospital‐based), study quality [higher quality (NOS ≥7) *versus* lower quality (NOS <7)] and language (Chinese *versus* English) to find the source of any heterogeneity.

Hardy–Weinberg equilibrium (HWE) was tested in control subjects in each study. Deviation from HWE was tested using the chi‐square test. Studies with controls that depart from HWE (*P* < 0.05) were subjected to a sensitivity analysis in order to check the consistency of the overall effect.

We made use of Begg's funnel plot to examine the underlying publication bias, and also used Egger's weighted regression method to calculate P for bias 26, 27. If no publication bias existed, the funnel plot looked symmetrical.

All analyses were conducted with the use of REVIEW MANAGER, V.5.2 (Revman, The Cochrane Collaboration) or STATA software, V.12.0 (STATA Corp, Lakeway Drive College Station, Texas, USA).

## Results

### Characteristics of included studies

**Figure 1:**
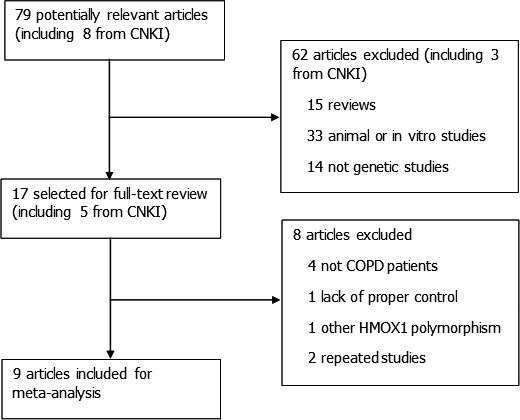
Study identification, inclusion and exclusion for meta‐analysis.

We identified 79 related articles, of which 17 studies were potentially suitable. Four studies were given up because objective population were not patients with COPD (one for lung cancer and others for general population). One study did not examine any *HMOX1* length polymorphism mentioned above. One study was excluded because of lack of proper control. Furthermore, two repeated studies were also ruled out. Thus, nine studies including 1447 cases and 891 controls met the including criteria (Fig. 1). *HOMX1* polymorphism was mentioned in seven studies, and four studies provided the association of *HMOX1* polymorphism with COPD severity. The study characteristics are listed in Tables 1 & 2. Patients with COPD were diagnosed through lung function index in all studies as well as radiography manifestation in some studies. General population and hospital‐based controls were involved in different studies. In addition, frequency‐matched controls to the cases by ethnicity, sex, age and smoking status were applied in some studies. Automated sequencer was applied to detect *HMOX1* genotypes in five of the nine studies. The scores of included studies ranged from 5 to 7 by NOS.

**Table 1:** Summary of studies about genetic association of *HMOX1* to COPD risk

Author	Year	Ethnicity	Sample Size		Source of control	Age matched	Smoking index matched	Lung function of COPD		COPD diagnosis	Genotype identification	NOS§
			Case	Control				FEV_1_/FVC%	FEV_1_/Pre%			
Budhi	2003	Asian	63	172	General population	No	Yes	Not mentioned	Not mentioned	Radiology and lung function†	Automated sequencing	5
Du	2006	Asian	64	56	General population	Yes	Yes	49.6 ± 7.0	53.6 ± 8.4	CMA guideline‡	PCR‐PAGE	7
Fu	2007	Asian	256	266	Hospital	Yes	Yes	48.2 ± 6.3	58.5 ± 10.0	GOLD##	Automated sequencing	6
Ma	2005	Asian	50	30	Hospital	Yes	Yes	Not mentioned	Not mentioned	CMA guideline	PCR‐PAGE	5
Matokanović	2012	Caucasian	130	95	General population	No	Yes	62.9 (54.9–67.1)*	41.5 ± 13.9	GOLD	Automated sequencing	7
Putra	2013	Asian	48	172	General population	No	Yes	Not mentioned	59 ± 15.6	GOLD	Automated sequencing	6
Yamada	2000	Asian	101	100	Hospital	Yes	Yes	47 ± 2	84 ± 7	Comprehensive***	Automated sequencing	7

*The data were presented as median (interquartile range).

†The patients were in accordance with early‐stage disease demonstrated by lung function test (the concrete data were not available) and radiology criteria as follows: chest CT indicated one with emphysematous changes showing low attenuation areas and/or bulla.

‡The guideline was published by Chinese Medical Association (CMA) for diagnosis and treatment of COPD in 2002.

##Global Initiative for Chronic Obstructive Lung Disease, the guideline for COPD diagnosis, management and prevention.

§Newcastle–Ottawa Scale, a tool for assess the quality of case–control study, ranged from 0 to 9.

***The patient with COPD was defined as a physical examination that demonstrated hyperresonant chest and flattened hemidiaphragms; a chest roentgenogram that demonstrated hyperinflation, flattened diaphragms and marked loss of vascularity; a computed tomography scan that demonstrated areas of low attenuation; and pulmonary function testing that demonstrated decreased FEV1:FVC ratios and impaired diffusion capacity

**Table 2:** Summary of studies about genetic association of *HMOX1* to COPD severity

Author	Year	Ethnicity	Less severe			More severe			COPD diagnosis	Genotype identification
			Sample Size	FEV_1_/FVC%	FEV_1_/Pre%	Sample Size	FEV_1_/FVC%	FEV_1_/Pre%		
Fu	2007	Asian	215	59.6 ± 10.4	76.6 ± 14.0	237	39.8 ± 9.3	37.5 ± 8.2	GOLD	Automated sequencing
Jiang	2006	Asian	60	46.3 ± 8.4	50.2 ± 10.0	45	25.9 ± 8.2	27.2 ± 7.6	CMA guideline	PCR‐PAGE
Matokanović	2012	Caucasian	37	Not mentioned	GOLD Stage II	93	Not mentioned	GOLD Stage III‐IV	GOLD	Automated sequencing
Putra	2013	Asian	25	Not mentioned	71.0 ± 5.8	23	Not mentioned	46.0 ± 10.8	GOLD	Automated sequencing

### *HMOX1* allele distribution in COPD and control groups

**Figure 2:**
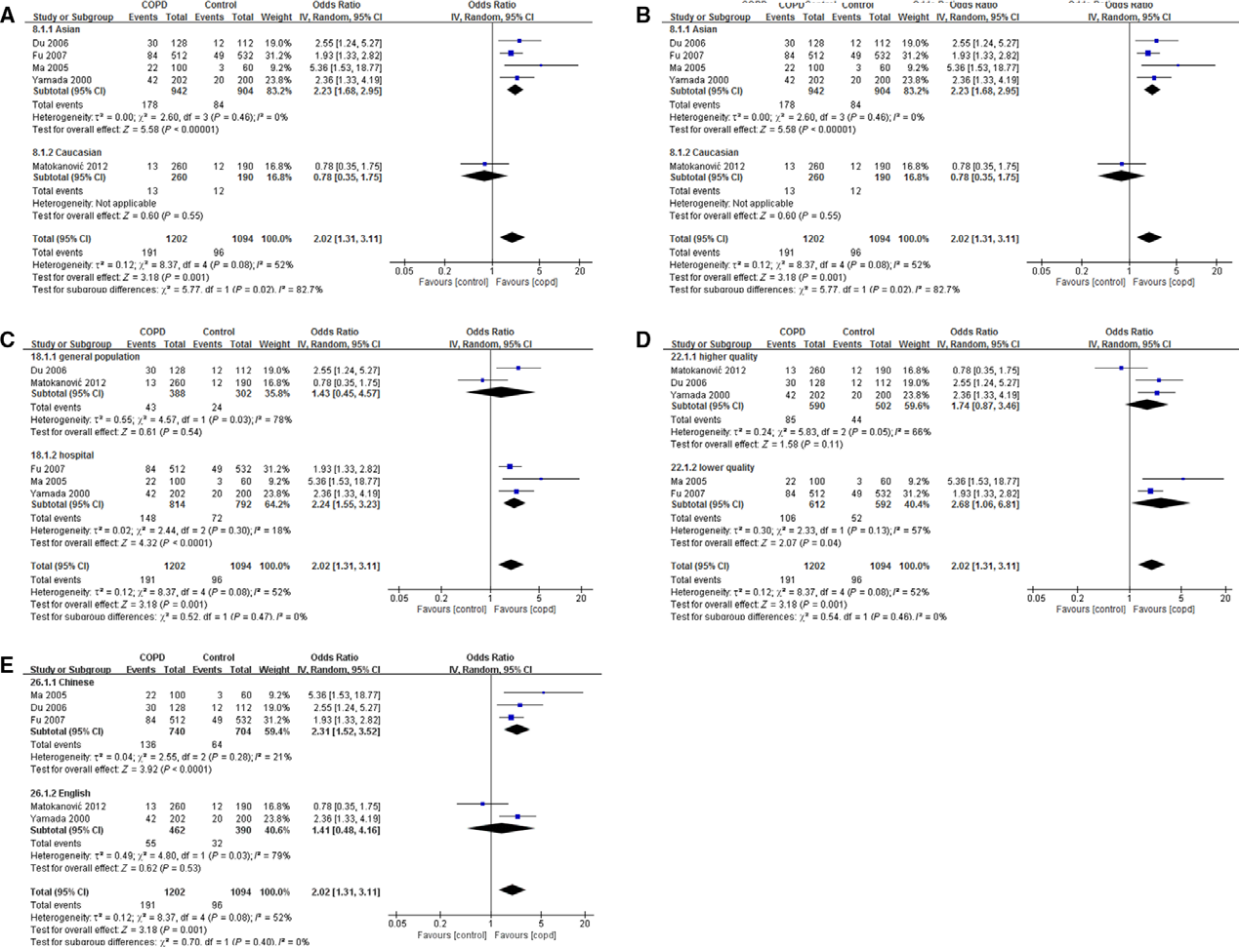
Correlation between L allele and COPD risk. The studies were divided into two groups according to ethnicity (Asian or Caucasian) (**A**), genotyping methods (automated sequencing or PCR‐PAGE) (**B**), source of control subjects (general population‐based or hospital‐based) (**C**), study quality (higher quality (NOS ≥7) or lower quality (NOS <7)) (**D**) and language (Chinese or English) (**E**) under the comparison of L *versus* S+M.

First, the distribution of each allele of *HMOX1* in both patients with COPD and control subjects was analysed with the use of random effect model. The frequencies of S and M allele were not different between these two groups. However, compared with control subjects, there was much higher frequency of L allele in patients with COPD (OR 2.02, 95% CI: 1.32–3.11, *P* = 0.001). Further analysis indicated that S but not M allele was a protective factor for COPD in Asian people (OR 0.62, 95% CI: 0.40–0.96, *P* = 0.03). Conversely, L allele suggested higher risk to COPD in this subpopulation (OR 2.23, 95% CI: 1.68–2.95, *P* < 0.001). Other than ethnicity, subgroup analysis also showed gene detection method, source of control subjects, the quality and language of included literature which could affect the results to some extent. Whereas less S alleles were observed in patients with COPD in PCR‐PAGE subgroup, more L carriers were found in patients with COPD in PCR‐PAGE, hospital‐based, lower quality and Chinese subgroups (Fig. 2).

### *HMOX1* genotypes distribution in COPD and control groups

**Figure 3:**
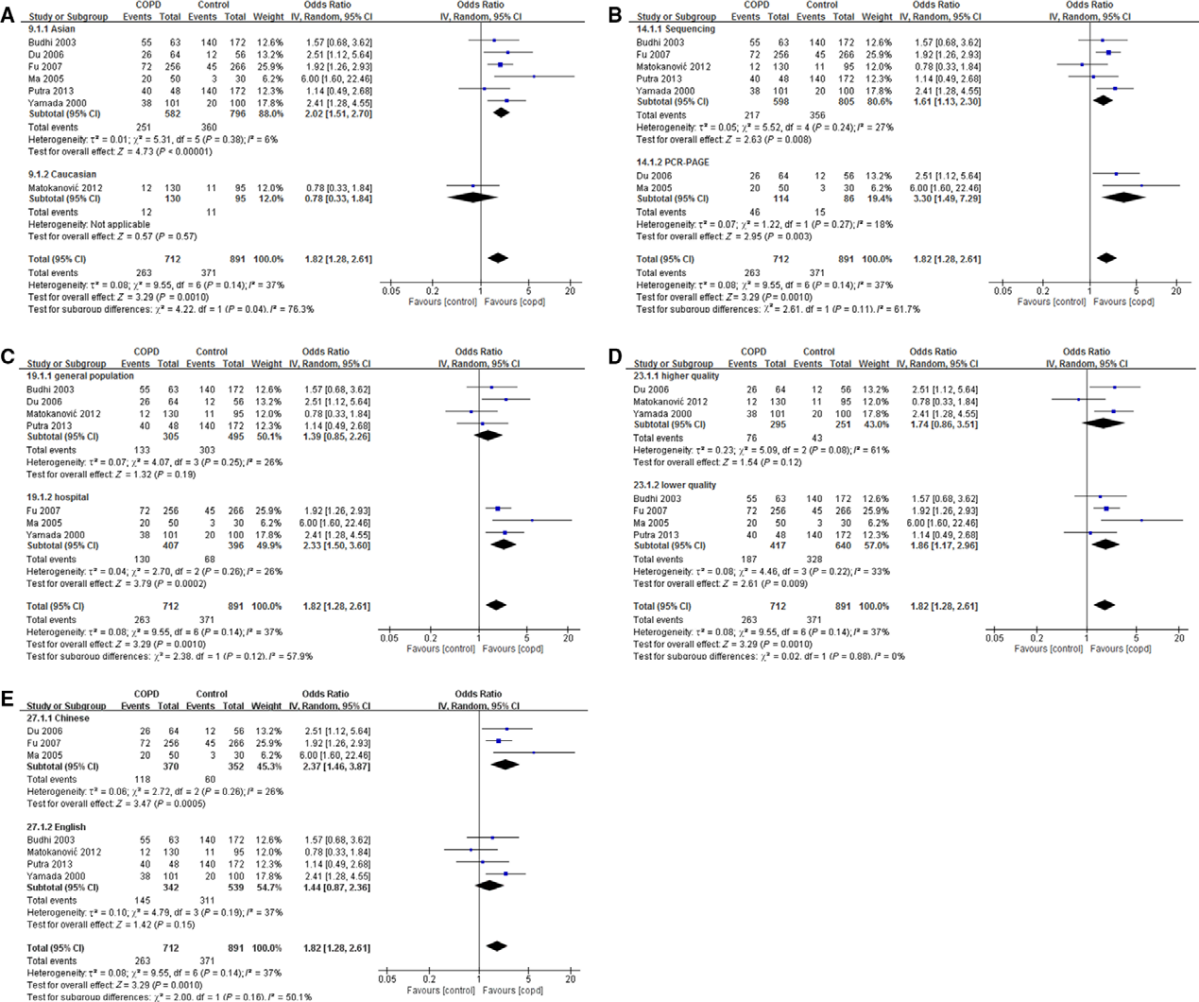
Correlation between *HMOX1* genotype and COPD risk. The studies were divided into two groups according to ethnicity (Asian or Caucasian) (**A**), genotyping methods (automated sequencing or PCR‐PAGE) (**B**), source of control subjects (general population‐based or hospital‐based) (**C**), study quality (higher quality (NOS ≥7) or lower quality (NOS <7)) (**D**) and language (Chinese or English) (**E**) under the comparison of type I *versus* type II.

*HMOX1* genotypes from seven studies are presented in Table 3. As a result, the proportion of type I genotype (L allele carrier) was higher than that of type II genotype (non‐L allele carrier) in patients with COPD (OR 1.82, 95% CI: 1.28–2.61, *P* = 0.001). Further analysis suggested that type I genotype was of dominant position in patients with COPD in several subgroups, including Asian (OR 2.02, 95% CI: 1.51–2.70, *P* < 0.001), hospital‐based (OR 2.33, 95% CI: 1.50–3.60, *P* < 0.001), lower quality (OR 1.86, 95% CI: 1.17–2.96, *P* = 0.009) and Chinese subgroup (OR 2.37, 95% CI: 1.46–3.87, *P* < 0.001). Moreover, in both subgroups divided by detection method, more L allele carriers were observed in patients with COPD than those in control subjects (for automated sequencing subgroup, OR 1.61, 95% CI: 1.13–2.30, *P* = 0.008; for PCR‐PAGE subgroup, OR 3.30, 95% CI: 1.49–7.29, *P* = 0.003; Fig. 3).

**Table 3:** the distribution of *HMOX1* alleles and genotypes in patients with COPD and control subjects

Author	Year	Allele						Genotype				HWE(*P*)
		Case			Control			Case		Control		
		S	M	L	S	M	L	Type I*	Type II†	Type I	Type II	
Budhi	2003	–	–	–	–	–	–	55	8	140	32	–
Du	2006	42	56	30	54	46	12	26	38	12	44	0.208
Fu	2007	233	195	84	243	240	49	72	184	45	221	<0.001
Ma	2005	29	49	22	31	26	3	20	30	3	27	0.172
Matokanović	2012	107	140	13	70	108	12	12	118	11	84	0.088
Putra	2013	–	–	–	–	–	–	40	8	140	32	–
Yamada	2000	67	93	42	92	88	20	38	63	20	80	0.103

*The subjects with at least one L allele.

†The subjects without L allele.

### *HMOX1* genotypes distribution in patients with COPD with different severity

Then, we further observed whether *HMOX1* genotypes were associated with COPD patients with different severity. Patients with COPD were divided into two groups according to their lung function. Unexpectedly, no evident difference of type I or type II genotype frequency was found in both groups (OR 0.97, 95% CI: 0.49–1.92). Due to the limited study numbers, subgroup analysis was not performed (Table S1).

### Heterogeneity and sensitivity analysis

For allele study, *I* ^2^ showed a distinct variation degree in different comparisons, from very low (M *versus* S+L) to quite high (S *versus* M+L) heterogeneity. In addition, moderate variation was found in genotype comparisons (Table 4). Subgroup analysis showed reduced heterogeneity in some subgroups in majority comparisons (Tables S2 & S3).

**Table 4:** Pooled odds ratio for COPD susceptibility and severity, heterogeneity and publication bias in meta‐analysis: comparison of alleles and genotypes

Comparison	Study number	OR [95% CI]	*P* value	Heterogeneity		Publication bias	
				*I* ^2^	P _heterogeneity_	Begg	Egger
COPD susceptibility							
S *versus* M+L	5	0.72 [0.49, 1.04]	0.08	76%	0.002	0.142	0.126
M *versus* S+L	5	0.91 [0.76, 1.10]	0.33	15%	0.32	0.142	0.021
L *versus* S+M	5	2.02 [1.31, 3.11]	0.001	52%	0.08	0.327	0.79
type I *versus* type II	7	1.82 [1.28, 2.61]	0.001	37%	0.14	0.453	0.949
COPD severity							
type I *versus* type II	4	0.97 [0.49, 1.92]	0.94	59%	0.06	0.497	0.335

Sensitivity analysis was conducted to assess the effect of each study on the overall results. Among these studies, Budhi *et al*. recruited patients with COPD with different inclusion criteria, whereas Ma *et al*. enrolled specific control subjects who were all lung cancer patients without airflow limitation. Moreover, the population in Fu's report was not in accordance with HWE. However, discarding these studies did not affect the pooled OR value in genotype comparisons, and neither did other studies (Fig. S1).

### Publication bias

**Figure 4:**
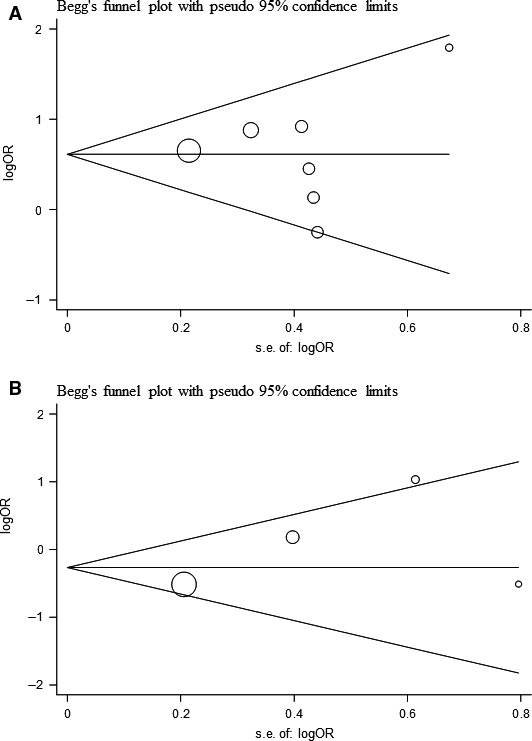
Publication bias on HMOX1 polymorphism. (**A**) Begg's funnel plot of the seven eligible studies of HMOX1 genotype distribution in COPD risk; (**B**) Begg's funnel plot of the four eligible studies of HMOX1 genotype distribution in COPD severity.

Publication bias was detected by Begg's and Egger's tests. These tests did not show significant results in almost all comparisons (Table 4). The funnel plots exhibited approximate symmetry shape (Fig. 4). These results indicated little publication bias.

## Discussion

As a complex disease, genetic background is considered to be another important factor related to COPD, other than environmental exposure 28. In the past years, genomewide association studies (GWAS) indicated several genes, including FAM13A (4q22), HHIP (4q31), CHRNA3/5 (15q25), IREB2 (15q25), MYO1D (17q11), VWA8 (13q14) and BICD1 (12p11), were related to COPD susceptibility 29, 30, 31, 32. However, heme oxygenase 1, a gene responsible for redox reactions in the microenvironment, has not been mentioned in these studies. There are several kinds of genetic polymorphisms, among which single nucleotide polymorphism (SNP) is the most common type. In our study, the genetic polymorphism we discussed is length polymorphism, which is the product of microsatellite DNA or short tandem repeat (STR) usually consisting of 2–6 repeated base pairs 33. GWAS mainly focused on SNP, but little attention has been paid to length polymorphism. Compared with SNP, the technique for accurate assessment of STR in whole genome is still under development 34. Moreover, the statistical models to analyse the association between STR phenotype and human disease in GWAS are considered to be defective 34. As a typical example of STR, previous researches suggested a long (GT)_n_ sequence in *HMOX1* promoter led to lower activity of antioxidation, which gave rise to the tendency to increased risk of COPD occurrence 19, 22, 35 and refractoriness to regular treatment 36, but there is still a lack of clear conclusion.

Our results indicated that L allele and type I genotype (L carriers) were the risk factors of COPD, especially for Asian population. These results were rather robust after sensitivity analysis. On the contrary, there was little association between *HMOX1* and COPD risk in Caucasians. It was in line with several GWAS, which were carried out in Western countries, and the enrolled subjects were largely Caucasians. Despite the ethnic difference, several reasons might be worthy to point out. First, there were no unified standard for classification of three kinds of alleles. For example, L allele was defined when the GT repeat number was equal to or more than 30 in Yamada's study 22, but this allele was not recognized unless the GT repeat number was larger than 31 in the Caucasian population 20. The inconsistent definitions of allele and genotype might cause the different results. Second, the inclusion criteria of patients with COPD were different among studies. In the report by Budhi *et al*. 14 the researches selected the patients whose disease were at early stage, which was quite different from other studies. Third, the selection of control subjects also need to note. In three studies, hospital‐derived control subjects were recruited to compare with COPD. Coincidently, positive results were observed in all these studies, especially in the study by Ma *et al*. 19. So selection bias might influence the overall effects.

In contrast to the association between HMOX1 and COPD occurrence, we did not find that HMOX1 (GT)_n_ polymorphism was related to COPD severity. Lung function is the most important index to assess the severity of COPD. So far, only a few studies have reported the relationship between HMOX1 polymorphism and lung function. Guenegou *et al*. found a long HMOX1 gene promoter was associated with accelerated decline in lung function in a general population, especially in heavy smokers 37, 38. In addition, the study by Nakayama *et al*. 39 revealed that the patients with COPD with L allele were at a much higher risk of rapid decrease in lung function than those without L allele. These results indicated that lung function was affected by HMOX1, which was observed in both Eastern and Western countries. However, our present study did not get positive result, and further research was needed to explain the discrepancy.

In our study, moderate to high heterogeneity existed in some comparisons, so subgroup analysis was introduced to seek out the source of heterogeneity. During the analysis, all the studies were divided into two subgroups according to ethnicity, source of control subjects, detection method, quality and language of literature. After stratification, one subgroup presented reduced or nearly disappeared heterogeneity in most comparisons, which demonstrated these factors were at least part source of heterogeneity. Among these factors, quality and language of literature were particularly noticeable because some papers were written in Chinese which might be considered as poor quality. However, there was little difference in quality between Chinese‐written papers and English‐written papers according to NOS score. As we did not set up language restriction of inclusion criteria, studies written in Chinese were also adopted into final analysis. In fact, we found in some subgroups, including Chinese subgroup, the role of L allele and genotype I as risk factor of COPD was reinforced. However, this phenomenon would rather be attributed to ethnicity rather than language. Compared with Chinese‐written studies, the subjects were mixed ethnicities in English‐written studies, including the one reported by Matokanovic *et al*. 20 which was the only study on Caucasian population. If this study was removed, positive results could be also observed in English subgroup. Nevertheless, it may remind us to distinguish confounder factors in future research.

There were some shortcomings in our work. First, the studies were limited in quantity and could not represent the whole population in the world. There were only about 1500 COPD subjects in these studies, which was a small part of patients with COPD worldwide. Moreover, the majority studies included in our present analysis were on Asian, and only one study mentioned Caucasian, to say nothing of Africans. Second, there were confounder factors in almost all the studies. As discussed above, the source of control subjects and method for genotype identification might amplify the main effect due to genetic factors. Last but not least, COPD is a chronic disease which is not fully reversible. So it is of significance to study the linkage between genotype and the degree of disease progression. However, we are lack of such data, which prevent us from further exploration.

In conclusion, we demonstrated L allele and genotype I were the risk factor of COPD, but not find *HMOX1* length polymorphism in promoter region was associated with COPD severity. Due to the various deficiencies in present studies, future studies with larger sample size, covering different populations, selecting proper control subjects and detection methods should be carried out to further validate the relationship between *HMOX1* and this disease.

## Conflict of interest

The authors report no conflict of interests.

## Author contribution

This study was designed by Zhou H and Li Y. The data were extracted by Ying X and Liu Y. Ye S and Yan J performed statistical analysis and graph drafting, respectively. This manuscript was originally written and finally approved by all the authors.

## Supporting information


**Figure S1** The influence of each study on overall result of COPD risk and severity
**Table S1** The distribution of *HMOX1* genotypes in COPD patients with different stages of severity
**Table S2** Subgroup analysis of association between *HMOX1* and COPD risk
