# Supplementary material for: Genetic polymorphism of heme oxygenase 1 promoter in the occurrence and severity of chronic obstructive pulmonary disease: a meta‐analysis
Source: J Cell Mol Med. 2016 Dec 20;21(5):894–903. doi: 10.1111/jcmm.13028 (PMC5387120; doi:10.1111/jcmm.13028)
Supplement: Supplementary file 1 — Figure S1 The influence of each study on overall result of COPD risk and severity Table S1 The distribution of HMOX1 genotypes in COPD patients with different stages of severity Table S2 Subgroup analysis of association between HMOX1 and COPD risk [file JCMM-21-894-s001.doc]

**Genetic Polymorphism of Heme Oxygenase1 Promoter in the Occurrence and Severity of Chronic Obstructive Pulmonary Disease: a Meta-analysis**

Hongbin Zhou^1^, Xiwang Ying^1^, Yuanshun Liu^1^, Sa Ye^1^, Jianping Yan^1^, Yaqing Li^1,*^

**Sensitivity analysis of each study related to *HMOX1* polymorphism and COPD**


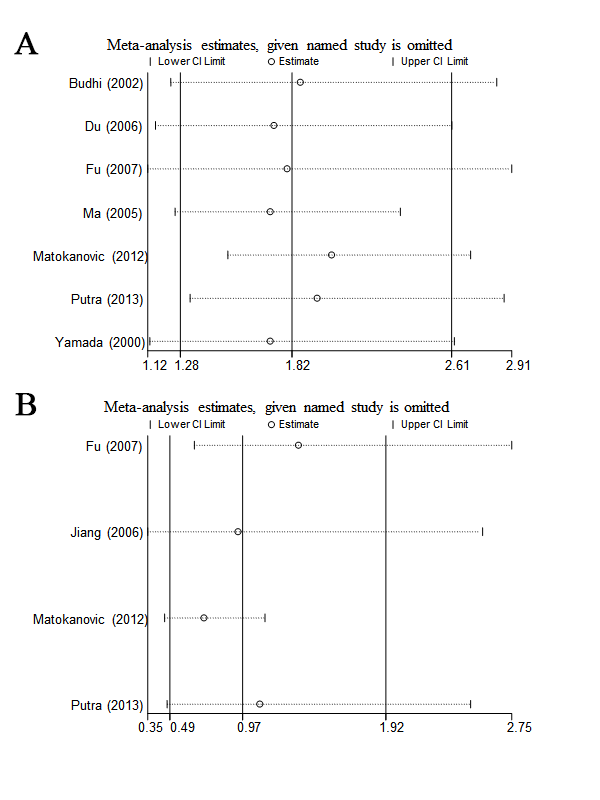


**Supplementary Figure S1** the influence of each study on overall result of COPD risk and severity. (A) Sensitivity analysis of the 7 eligible studies of *HMOX1* genotype distribution in COPD risk; (B) Sensitivity analysis of the 4 eligible studies of HMOX1 genotype distribution in COPD severity.

**Supplementary Table S1** the distribution of *HMOX1* genotypes in COPD patients with different stages of severity

| Author | Year | Less severe | |  | More severe | |
| --- | --- | --- | --- | --- | --- | --- |
|  |  | **Type Ⅰ*** | **Type Ⅱ**** |  | **Type Ⅰ** | **Type Ⅱ** |
| Fu | 2007 | 56 | 159 |  | 88 | 149 |
| Jiang | 2006 | 28 | 32 |  | 19 | 26 |
| Matokanović | 2012 | 6 | 31 |  | 6 | 87 |
| Putra | 2013 | 20 | 5 |  | 20 | 3 |

* The subjects with at least one L allele

** The subjects without L allele

**Supplementary Table S2** Subgroup analysis of association between *HMOX1* and COPD risk

| subgroup |  | Allele comparison | | | | | | | | | | | | | | |  | | Genotype comparison | | |
| --- | --- | --- | --- | --- | --- | --- | --- | --- | --- | --- | --- | --- | --- | --- | --- | --- | --- | --- | --- | --- | --- |
|  |  | **S vs. M+L** | | | |  | **M vs. S+L** | | | |  | **L vs. S+M** | | | |  | | **type Ⅰ vs. type Ⅱ** | | | |
|  |  | Study number | | OR  [95% CI] | I^2^ |  | Study  number | | OR  [95% CI] | I^2^ |  | Study number | | OR  [95% CI] | I^2^ |  | | Study number | | OR  [95% CI] | I^2^ |
| **Ethnicity** | | | | | | | | | | | | | | | | | | | | | |
| Asian |  | 4 | 0.62  [0.40, 0.96]* | | 76% |  | 4 | 0.95  [0.73, 1.23] | | 36% |  | 4 | 2.23  [1.68, 2.95]** | | 0 |  | | 6 | | 2.02  [1.51, 2.70]** | 6% |
| Caucasian |  | 1 | 1.20  [0.82, 1.76] | | NA# |  | 1 | 0.89  [0.61, 1.29] | | NA# |  | 1 | 0.78  [0.35, 1.75] | | NA# |  | | 1 | | 0.78  [0.33, 1.84] | NA# |
| **Genotyping method** | | | | | | | | | | | | | | | | | | | | | |
| Automated sequencing |  | 3 | 0.90  [0.62, 1.30] | | 72% |  | 3 | 0.86  [0.69, 1.06] | | 22% |  | 3 | 1.67  [0.99, 2.81] | | 61% |  | | 5 | | 1.61  [1.13, 2.30]** | 27% |
| PCR-PAGE |  | 2 | 0.46  [0.31, 0.70]** | | 0 |  | 2 | 1.17  [0.78, 1.75] | | 0 |  | 2 | 3.08  [1.64, 5.79]** | | 1% |  | | 2 | | 3.30  [1.49, 7.29]** | 18% |
| **Source of control** | | | | | | | | | | | | | | | | | | | | | |
| General population |  | 2 | 0.81  [0.36, 1.82] | | 84% |  | 2 | 0.96  [0.71, 1.30] | | 0 |  | 2 | 1.43  [0.45, 4.57] | | 78% |  | | 4 | | 1.39  [0.85, 2.26] | 26% |
| Hospital |  | 3 | 0.65  [0.38, 1.10] | | 80% |  | 3 | 0.93  [0.68, 1.28] | | 49% |  | 3 | 2.24  [1.55, 3.23]** | | 18% |  | | 3 | | 2.33  [1.50, 3.60]** | 26% |
| **Literature quality** | | | | | | | | | | | | | | | | | | | | | |
| Higher quality |  | 3 | 0.73  [0.43, 1.24] | | 78% |  | 3 | 1.01  [0.79, 1.28] | | 0 |  | 3 | 1.74  [0.87, 3.46] | | 66% |  | | 3 | | 1.74  [0.86, 3.51] | 61% |
| Lower quality |  | 2 | 0.65  [0.26, 1.65] | | 86% |  | 2 | 0.89  [0.55, 1.43] | | 54% |  | 2 | 2.68  [1.06, 6.81]* | | 57% |  | | 4 | | 1.86  [1.17, 2.96]** | 33% |
| **Language** | | | | | | | | | | | | | | | | | | | | | |
| Chinese |  | 3 | 0.62  [0.34,1.13] | | 80% |  | 3 | 0.93  [0.66, 1.30] | | 43% |  | 3 | 2.31  [1.52, 3.52] | | 21% |  | | 3 | | 2.37  [1.46, 3.87] | 26% |
| English |  | 2 | 0.84  [0.41, 1.70] | | 84% |  | 2 | 0.98  [0.74, 1.28] | | 0 |  | 2 | 1.41  [0.48, 4.16] | | 79% |  | | 4 | | 1.44  [0.87, 2.36] | 37% |

# Not applicable

* p < 0.05

** p < 0.01
